# Potassium deficiency diagnosis method of apple leaves based on MLR-LDA-SVM

**DOI:** 10.3389/fpls.2023.1271933

**Published:** 2023-11-29

**Authors:** Kun Xu, Lin-Lin Sun, Jing Wang, Shuang-Xi Liu, Hua-Wei Yang, Ning Xu, Hong-Jian Zhang, Jin-Xing Wang

**Affiliations:** ^1^ College of Mechanical and Electrical Engineering, Shandong Agricultural University, Taian, China; ^2^ Shandong Academy of Agricultural Machinery Science, Shandong Academy of Agricultural Sciences, Jinan, China; ^3^ Shandong Provincial Key Laboratory of Horticultural Machinery and Equipment, Shandong Agricultural University, Taian, China; ^4^ Shandong Agricultural Equipment Intelligent Engineering Laboratory, Shandong Agricultural University, Taian, China

**Keywords:** apple leaves, potassium deficiency, diagnostic method, support vector machine, shape-color feature

## Abstract

**Introduction:**

At present, machine learning and image processing technology are widely used in plant disease diagnosis. In order to address the challenges of subjectivity, cost, and timeliness associated with traditional methods of diagnosing potassium deficiency in apple tree leaves.

**Methods:**

The study proposes a model that utilizes image processing technology and machine learning techniques to enhance the accuracy of detection during each growth period. Leaf images were collected at different growth stages and processed through denoising and segmentation. Color and shape features of the leaves were extracted and a multiple regression analysis model was used to screen for key features. Linear discriminant analysis was then employed to optimize the data and obtain the optimal shape and color feature factors of apple tree leaves during each growth period. Various machine-learning methods, including SVM, DT, and KNN, were used for the diagnosis of potassium deficiency.

**Results:**

The MLR-LDA-SVM model was found to be the optimal model based on comprehensive evaluation indicators. Field experiments were conducted to verify the accuracy of the diagnostic model, achieving high diagnostic accuracy during different growth periods.

**Discussion:**

The model can accurately diagnose whether potassium deficiency exists in apple tree leaves during each growth period. This provides theoretical guidance for intelligent and precise water and fertilizer management in orchards.

## 1 Introduction

China, known as one of the most significant apple native centers in the world (Shu and Zhang, 2021), holds the top position in global apple production and planting areas. In 2022, China’s total apple production increased by 3.4% compared to 2021, reaching 47.571 million tons. Potassium is an essential nutrient element for the growth and development of apple trees (Wang et al., 2019). It plays a crucial role in enhancing leaf photosynthesis, promoting fruit enlargement, and increasing fruit weight per fruit. Potassium deficiency in fruit trees can result in yellow leaves, shriveling and curling, poor fruit development, small size, reduced sugar content, poor color, and weak taste (Wang, 2023; Chen and Li, 2018; Wei et al., 2022). Therefore, accurately diagnosing potassium deficiency in fruit trees is significant in guiding increased yield and improved fruit quality. Leaf K content is positively correlated with K content in branches and shoots of fruit trees (Peng, 2016). Nutritional diagnosis analysis based on leaves can serve as the basis for nutritional analysis and diagnosis of apple trees. Traditional diagnostic methods for crop potassium deficiency include empirical diagnosis and chemical determination (Ullah et al., 2022). Empirical diagnosis is subjective and has significant limitations, which can lead to human errors. Although chemical determination methods yield accurate results, they present challenges such as requiring professional operation, expensive equipment, and poor timeliness. In recent years, with the rapid development of computer technology, image processing technology has been widely used in the field of crop nutrient deficiency diagnosis due to its simplicity, affordability, and speed.

Researchers have made significant progress in diagnosing crop nutrient deficiencies using machine learning and hyperspectral technology. In the field of machine learning, various algorithms have been used to predict nutrient deficiencies in different crops. For example, by analyzing color characteristics, the K-nearest neighbor algorithm has been successfully applied to predict potassium deficiency in grape and mango leaves. This involves segmenting and extracting yellows in leaf edges and tips (Rangel et al., 2016; Merchant et al., 2018). Another approach is the use of a BP neural network optimized by a genetic algorithm, which has achieved an impressive average accuracy of 99% in diagnosing nitrogen deficiency in rice treated with different nitrogen treatments (Luo et al., 2020). Decision tree algorithms have also been utilized to classify rice samples with diverse nutrient levels, resulting in highly accurate outcomes (Shi, 2011). Deep convolutional neural networks, such as the Inception-ResNet V2 and Autoencoder algorithm models, have been employed to identify and predict nutritional defects in tomato leaves and fruits, leading to improved diagnostic accuracy. This approach has also provided insights into identifying tomato diseases caused by nutrient deficiencies (Jae-Won et al., 2018). Furthermore, a non-destructive and rapid method for detecting grape leaf potassium content has been developed using the CNN-M1 model, which analyzes image color components and combination parameters, as well as the correlation between image features and leaf potassium content (Yang et al., 2021). Digital image technology has also been utilized to analyze the correlation between the G/(R+G+B) linear equation model of the Chinese cabbage canopy image and nitrogen nutrition indexes, offering promising ideas for nitrogen nutrition diagnosis (Li et al., 2022). The LeNet-5 model has been applied to classify three different diseases of apple leaves with an accuracy of 92% (Zheng, 2022). Support vector machine classifiers have been used to diagnose and analyze common deficiencies in rape, achieving an overall accuracy of 93% (Zhang et al., 2016). Spectral monitoring has also yielded promising results: multi-spectral reflection imaging data used to quantify the growth periods of maize and identify symptoms of potassium deficiency in maize leaves through a linear regression model, verifying the feasibility of multi-spectral monitoring (Furlanetto et al., 2021). The GA-IPLS model has been employed to establish the correlation between cucumber leaf hyperspectral image data and leaf chlorophyll content, proposing a potassium deficiency diagnosis method based on chlorophyll distribution characteristics with an accuracy of 95% (Shi et al., 2019; Shi et al., 2021). Reflectance spectrum and hyperspectral imaging have been used to predict the phosphorus level of rice through the establishment of a spectral curve of rice phosphorus (Takehisa et al., 2022). A universal wheat single-leaf potassium monitoring model has been established by analyzing and comparing the spectral variation characteristics of wheat single-leaf slices under different potassium nutrient conditions (Qi, 2017). The rice canopy spectral information obtained by hyperspectral remote sensing of an unmanned aerial vehicle has been used to establish a normalized difference BA-ELM model, enabling the construction of the critical nitrogen concentration curve of rice in Northeast China and realizing the nitrogen nutrition diagnosis of rice in the region (Xu et al., 2023). Additionally, a cotton nitrogen spectrum monitoring model has been established to monitor cotton nitrogen levels through the exploration of the relationship between nitrogen content in cotton leaves and the multi-angle hyperspectral data (Zhou et al., 2023). This research demonstrates the potential of machine learning and hyperspectral technology in identifying and diagnosing crop diseases resulting from nutrient deficiencies.

In comparison to image processing analysis for diagnostics, spectral analysis offers the advantage of providing accurate results. However, its widespread adoption is hindered by challenges such as high costs and the need for bulky equipment. In the current state of research, both domestically and internationally, data image technology has found extensive applications in crop nutrient diagnostics. However, there is a lack of research on personalized nutrient diagnostics in apple trees. To address this gap, this study employed digital image processing and machine learning techniques to achieve a precise diagnosis of potassium deficiency in apple tree leaves. Various color and shape features were extracted from apple tree leaves at different growth stages, and influential factors were analyzed to identify key features. The study determined the optimal shape and color feature factors and developed a potassium deficiency diagnosis model for apple tree leaves based on MLR-LDA-SVM. This research provides theoretical guidance for intelligent and precise water and fertilizer management in orchards.

## 2 Materials and methods

### 2.1 Sample collection area

**Figure 1:**
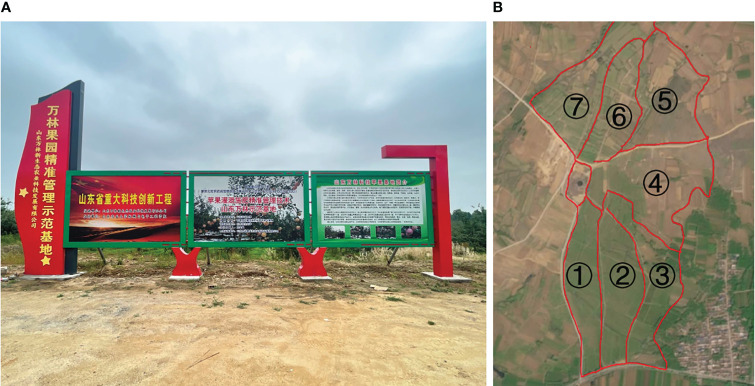
Wanlin Apple Experimental Park. **(A)** Park diagram. **(B)** Seven areas diagram.

This study obtained apple leaf samples from the Wanlin Orchard Precision Management Demonstration Base, located in Geshi Town, Ningyang County, Tai’an City, Shandong Province (116° 49’e, 35° 45’n). The base comprises seven areas (**Figure 1**), predominantly characterized by low mountains and hills, with an average elevation of 69.2 meters, average annual precipitation of 687.2mm, and average annual sunshine of 2627.1h. Red Fuji, a prevalent apple species, was selected for the investigation. Specifically, apple tree leaf samples were collected from the second area for method training and testing. In contrast, samples from the third area were collected to verify the method’s performance in the field experiment. The collection comprised 160 six-year-old apple trees with uniform growth in the second plot, each labeled with a land tag numbered 1-170. The fruit trees were in the full fruit periods, with an average height of 3.0m and an average crown width of 3.5m.

Before formal sample collection, the soil in the experimental area was analyzed for essential nutrient content and pH. Results showed that the soil had an organic matter content of 1.59%, total nitrogen content of 0.89%, available phosphorus content of 48.4mg/kg, available potassium content of 24.7mg/kg, available zinc content of 2.5mg/kg, available boron content of 0.8mg/kg, available iron content of 9.3mg/kg, and a pH of 6.3. These measurements classified the soil as slightly acidic brown soil according to the national soil nutrient classification standard. The content of organic matter, total nitrogen, available phosphorus, and available zinc in the soil was at the first level. In contrast, available boron and iron content was classified as the third level. The content of available potassium was shallow, classified as grade 6. These results indicated that the experimental area met the apple tree leaf sample collection requirements.

### 2.2 Formulation of fertilization methods

To obtain apple leaf samples with different potassium content in each growth period, artificial fertilization interventions were conducted in the second area of the experimental site. To ensure consistency in flower thinning, fruit thinning, pruning, nitrogen and phosphate fertilizer application, irrigation amount, trace element application (Tian, 2022), and drug application for all 170 experimental apple trees throughout the growth cycle, different gradients of potassium fertilizer application programs were designed for each growth period based on tree age, yield, and soil nutrient levels. Labels 1-50 received no K treatment, labels 50-90 received low K application (0.3), labels 91-130 received low-medium K application (0.6), and labels 131-170 served as the control group (**Table 1**).

**Table 1:** Gradient fertilization scheme for each growth period.

Period	Treatment	Fertilization program (Per plant/g) whole year: N: P2O5:K2o=2:1:2		
		Urea (46%N)	Diammonium hydrogen phosphate (18% N, 46% P2O5)	potassium sulphate (50%K_2_O)
Before germination (Early March)	K_0_ K_0.3_ K_0.6_ K_1_	63	39	0
Promote fruit (Late May)	K_0_ K_0.3_ K_0.6_ K_1_	24	39	0 24 48 72
Fruit expansion period (Early July)	K_0_ K_0.3_ K_0.6_ K_1_	32	19	0 60 120 180
Post-harvest (Early November)	K_0_ K_0.3_ K_0.6_ K_1_	196	98	0 36 72 108

### 2.3 Platform and environment

The software environment of this experiment was Windows 10 (64-bit) system, and Python was used as the programming language to complete sample image segmentation, image de-lighting, and image feature extraction. The Scikit-learn open-source machine learning framework was used to realize MLR, LDA, SVM, DT, KNN, and other methods. The hardware environment was equipped with Intel(R) Xeon(R) Silver 4210R CPU @ 2.40GHz 2.39GHz processor. The machine belt memory is 64GB. The camera Canon EOS 80D was used for the test image acquisition. The image size was 6000 pixels ×4000 pixels, the focal length was 35mm, and natural light was used.

### 2.4 Data acquisition

#### 2.4.1 Sample collection and leaf image acquisition

Leaves were systematically collected at various growth periods of fruit tree No. 1-170, namely April 13 and May 12 (flowering period), June 16 and July 10 (young fruit period), July 23 and August 12 (fruit enlarging period), as well as September 16 and September 30 (mature period). The collection was conducted 15 days after topdressing at each growth period. To ensure the experiment’s validity, two fresh leaves with similar growth, complete leaves, and free from any noticeable pests or diseases were meticulously handpicked from each of the ten different parts of the fruit tree.

**Figure 2:**
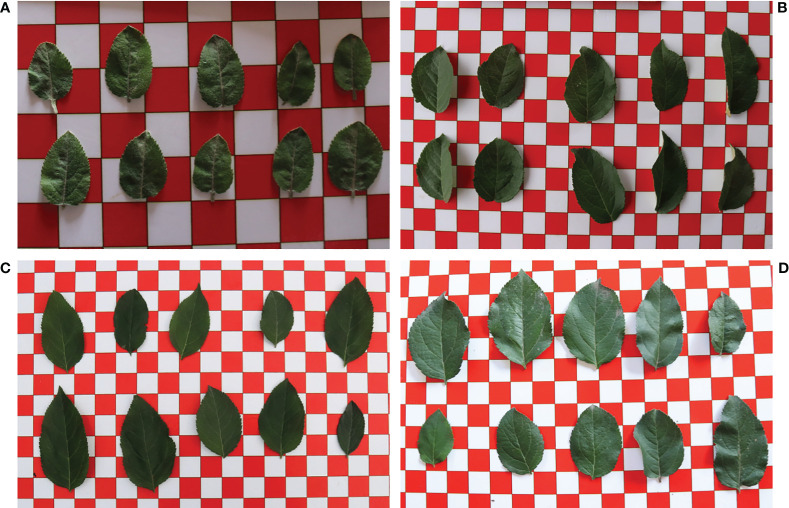
Images of apple leaves at each growth period. **(A)** Flowering period. **(B)** Young fruit period. **(C)** Fruit enlarging period. **(D)** Mature period.

To maintain consistency and traceability, the samples collected from the same fruit tree were assembled and then packed into appropriately labeled envelopes. To eliminate any potential external interference, the dust and stains on the surface of the leaves were delicately washed with clean water, and the excess surface moisture was dried with absorbent paper. Ten leaves were randomly selected from each group as test samples and were placed on a 2×2cm optical calibration board. A Canon EOS 80D camera then captured the leaf images under natural light conditions. Subsequently, the leaves were returned correctly to their respective envelopes and transported to the laboratory. Finally, the leaf images of the same fruit tree in each growth period were obtained (**Figure 2**).

#### 2.4.2 Chemical determination of total potassium content in leaves

**Figure 3:**
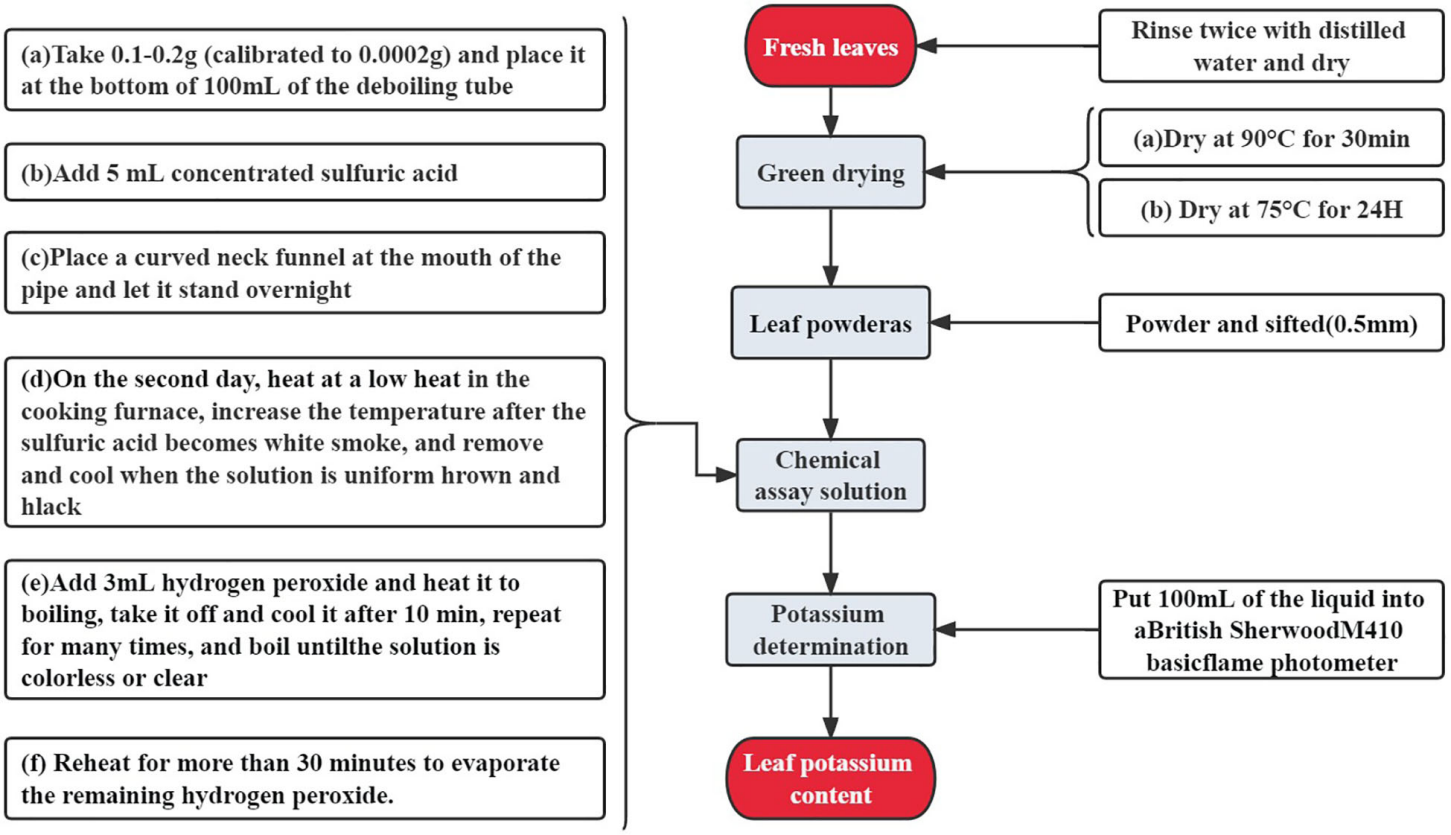
Flow chart of chemical determination of total potassium content in leaves.

The potassium content of apple leaves was quantified using flame photometry. The fresh leaves were prepared carefully and processed according to the flow chart (**Figure 3**) to obtain the exact potassium content.

In line with the mineral element content grading standard for apple trees, the leaves were categorized based on either potassium deficiency or normal potassium levels (**Table 2**).

**Table 2:** Potassium deficiency and the average number of fruit trees after artificial intervention.

Period	K_0_		K_0.3_		K_0.6_		K_1_	
	deficiency	normal	deficiency	normal	deficiency	normal	deficiency	normal
Flowering period	40	10	18	22	8	32	0	40
Young fruit period	48	2	28	12	20	20	0	40
Fruit enlarging period	50	0	40	0	38	2	0	40
Mature period	50	0	40	0	40	0	2	38

### 2.5 Leaf image preprocessing

**Figure 4:**
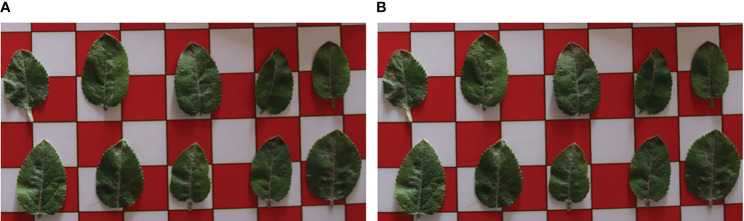
Comparison before and after Gaussian filter denoising. **(A)** Original image. **(B)** Gaussian filter denoising image.

To mitigate noise, enhance image quality, and accentuate edge information, a Gaussian filter was applied to the leaf images to achieve image denoising (Gao et al., 2020). The contrast between the denoised and original images is improved (**Figure 4**).

**Figure 5:**
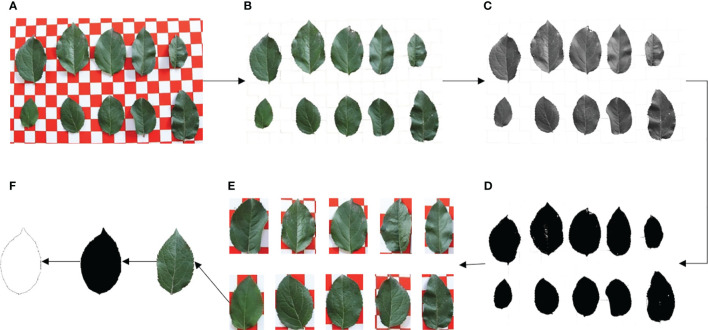
Blade pretreatment flow chart. **(A)** Image after Gaussian filtering. **(B)** Leaves reserved. **(C)** Image decolorization. **(D)** Image binarization. **(E)** Single leaf segmentation. **(F)** Blade contour extraction.

To streamline the subsequent extraction of leaf information, the denoised whole leaf image was separated into individual leaves. The background of the calibration plate was eliminated using the threshold segmentation method, while the Canny operator was utilized for edge monitoring to extract the leaf contour (**Figure 5**) (Fuentes-Alventosa et al., 2022).

**Figure 6:**
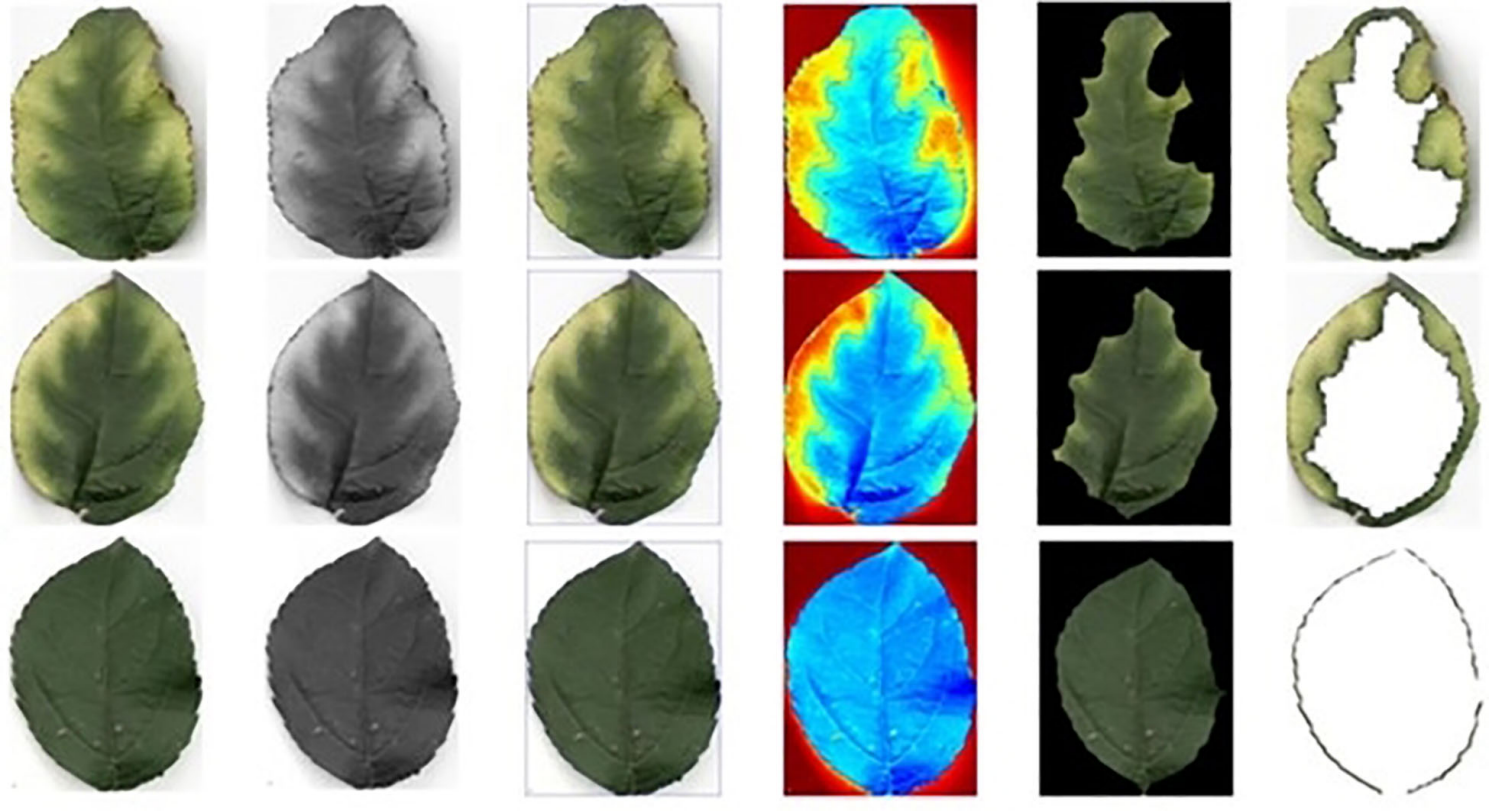
Image after segmentation of leaf margin yellowing area.

During the subsequent shape feature extraction, it is crucial to use specific parameters for the leaf edge yellowing area and leaf spot area to ensure accurate segmentation of these two areas. The green area is more concentrated and uniform than the yellow area. Therefore, the green portion of the individual leaf was selected for extraction, and the original leaf was subtracted to obtain the yellowing area along the leaf edge (**Figure 6**).

**Figure 7:**
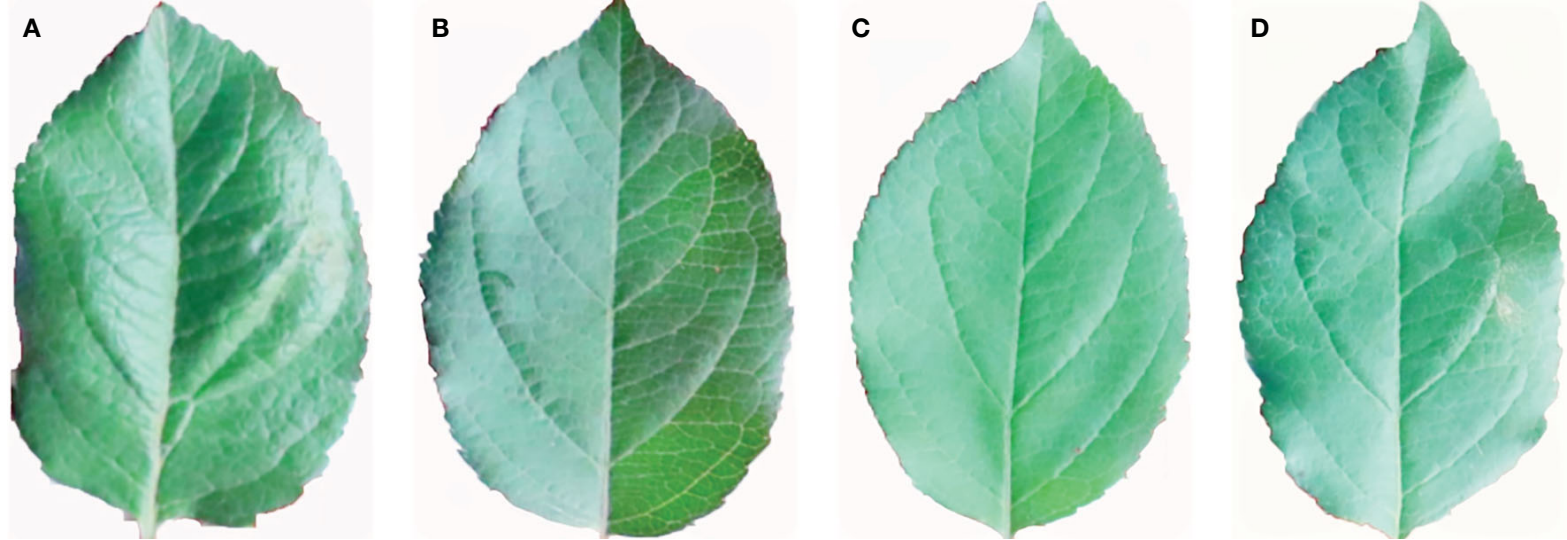
Leaves MSRCR de-lighting at each growth period. **(A)** Flowering period. **(B)** Young fruit period. **(C)** Fruit enlarging period. **(D)** Mature period.

The leaves were collected under natural light conditions (**Supplementary Figure 1**). To avoid the impact of uneven local illumination and varying image brightness caused by weather and occlusion on the image extraction performance, the MSRCR algorithm (Multi-Scale Retinex with color restoration) was employed to adjust the image illumination (Wang et al., 2018; Zheng et al., 2019). The resulting leaf images at each period after illumination adjustment were obtained (**Figure 7**). The local contrast of the image was enhanced, while the illumination was made uniform, and the brightness of the images was similar to that observed under actual conditions.

### 2.6 Leaf image feature extraction

#### 2.6.1 Leaf image feature extraction

**Figure 8:**
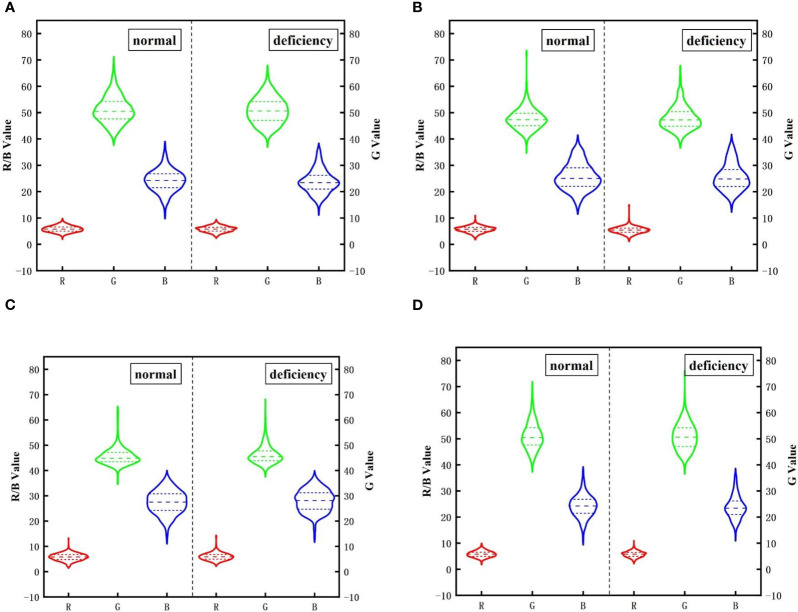
Mean distribution of RGB monochromatic components of potassium in different growth periods. **(A)** Flowering period. **(B)** Young fruit, period. **(C)** Fruit enlarging period. **(D)** Mature period.

The color change of apple leaves is a primary indicator of early-period potassium deficiency in apple trees. When potassium content is moderate, the leaves appear dark green and vibrant. However, when the potassium levels are low, the leaves tend to show yellowing, browning, and scorching. Brown spots and patches may also appear, while the veins remain green. In severe cases of potassium deficiency, the entire leaves may appear reddish-brown or dry. In this study, the mean values of R, G, B, H, S, V, L, A, and B monochromatic components of leaves at each growth period were extracted in RGB, HSV, and LAB color spaces. The color characteristics were further expanded using NRI (red light standard value), NGI (green light standard value), and NBI (blue light standard value) commonly used in leaf research. The average distribution of monochromatic components of typical and potassium-deficient apple leaves after pretreatment in RGB space was compared (**Figure 8**). Meanwhile, the mean values of monochromatic components in HSV (**Table 3**) and LDA (**Supplementary Table 1**) Spaces are calculated. It is evident from the figure that there are notable differences in the color characteristics of typical and potassium-deficient leaves at each growth period.

**Table 3:** Mean value of leaf HSV monochromatic component in each growth period.

Period	Nutrition	H			S			V		
		MAX	MIN	AVG	MAX	MIN	AVG	MAX	MIN	AVG
Flowering period	deficiency	85.11	63.33	72.39	245.5	201.4	225.7	70.13	39.21	51.13
	normal	88.74	63.02	72.32	243.5	183.2	225.7	74.73	37.98	50.81
Young fruit period	deficiency	90.49	65.31	74.51	264.9	149.8	225.8	72.42	35.57	47.61
	normal	91.49	63.98	74.12	243.7	193.5	224	64.61	38.76	47.93
Fruit enlarging period	deficiency	88.29	63.54	76.24	246.2	168.1	222.7	67.84	38.19	46.25
	normal	85.78	60.8	76.65	239.3	157.1	221.4	64.02	35.86	45.57
Mature period	deficiency	88.03	62.13	75.73	244.8	183.7	224.9	69.48	38.68	46.39
	normal	85.99	65.89	76.9	24.08	190.5	224.7	66.18	39.18	45.43

#### 2.6.2 Leaf image shape feature extraction

Apart from color features, there were also substantial differences in leaf shape features between regular and potassium-deficient apple trees. When potassium was lacking, the growth rate of the tree body was slower, and the leaf margin tended to curl up. The new leaves were generally smaller, while the old leaves gradually became necrotic. Therefore, a wide range of shape features were extracted from the whole and local leaves. These features were combined with color features and applied to diagnose potassium deficiency.

Following binarization, contour extraction, and segmentation of leaf discolor regions, the geometric parameters of leaf characteristics, such as long axis (*l_1_*), short axis (*l_2_*), perimeter (*C*), leaf area (*S*), leaf edge discolor area (*S_1_*), and leaf spot area (*S_2_*) were computed (**Supplementary Figure 2**).

By using the above characteristic geometrical parameters, the four shape features of eccentricity (E), shape parameter (F), color change ratio (CR), and spot change ratio (SR) can be deduced.

### 2.7 Method construction

#### 2.7.1 Multiple linear regression

Multiple Linear Regression (MLR) is a widely used statistical method (Korkmaz, 2021). It is commonly employed to establish quantitative descriptions of the linear dependencies between dependent and multiple independent variables. The least squares method is utilized to identify a curve that minimizes the sum of Euclidean distances from all samples to the line (Etemadi and Khashei, 2021).

#### 2.7.2 Linear discriminant analysis

Linear Discriminant Analysis (LDA) is a widely used supervised dimensionality reduction algorithm (Zhang et al., 2019; Gardner-Lubbe, 2021). The basic idea is to project a given training data set onto a straight line so that the projection points of similar data are as close as possible. The projection points of heterogeneous data are as far away as possible to achieve the effect of extracting classification information and compressing the feature space dimension. The core of this algorithm is to find the best projection direction that can best distinguish the data between classes so that the intra-class contrast is slight and the between-class mean difference is significant.

#### 2.7.3 Support vector machine classifier

Support vector machine (SVM) is a machine learning algorithm based on statistical learning theory (Guenther and Schonlau, 2016). It is a generalized linear classifier that performs binary classification based on supervised learning and possesses exceptional generalization ability for unobserved samples. The fundamental concept of SVM is identifying an optimal separation hyperplane in the sample space using the training set to segregate samples of different classes. Suppose SVM is applied to tackle multi-class classification issues. In that case, a combination principle must be utilized to establish a multi-class classifier based on binary classification, followed by implementing multi-class classification. SVM classifiers offer several advantages, including solid generalization ability, the ability to handle high-dimensional datasets, and the ability to address minor sample size problems.

#### 2.7.4 K-nearest neighbor classifier

K-nearest neighbor (KNN) is a frequently used supervised learning method (Jiang et al., 2012). Its working mechanism is relatively straightforward: Given a test sample, the K nearest training samples in the training set are identified based on some distance metric. Then, a prediction is made based on these K “neighbors” information.

#### 2.7.5 Decision tree classifier

Decision tree (DT) is a prevalent machine-learning technique (Wang et al., 2020). It consists of a root node, various internal nodes, and several leaf nodes. The leaf node corresponds to the decision outcome, and each other node corresponds to an attribute test. The sample set contained in each node is subdivided into child nodes based on the outcome of the attribute test, and the root node contains the entire sample set.

## 3 Results and analysis

This paper established an accurate diagnosis method for potassium deficiency in apple leaves using machine learning algorithms. The dataset was optimized through MLR and LDA, and SVM, DT, KNN, and other algorithms were employed to accurately classify potassium-deficient and regular leaves. Four performance indicators, namely accuracy (*Acc*), Recall (*Rec*), Precision (*Pre*), and F1-Score (*F1*), were utilized to evaluate the method and determine the optimal diagnostic method for potassium deficiency in apple leaves.

### 3.1 Data set optimization

The entire dataset was divided into two subsets: one with a specific potassium content and the other lacking a specific potassium content. For the subset of samples with a specific potassium content, a Multiple Linear Regression (MLR) method was established, enabling the identification of influential factors and their respective average values. The average value from the table (**Supplementary Table 2**) is 0.35.

**Figure 9:**
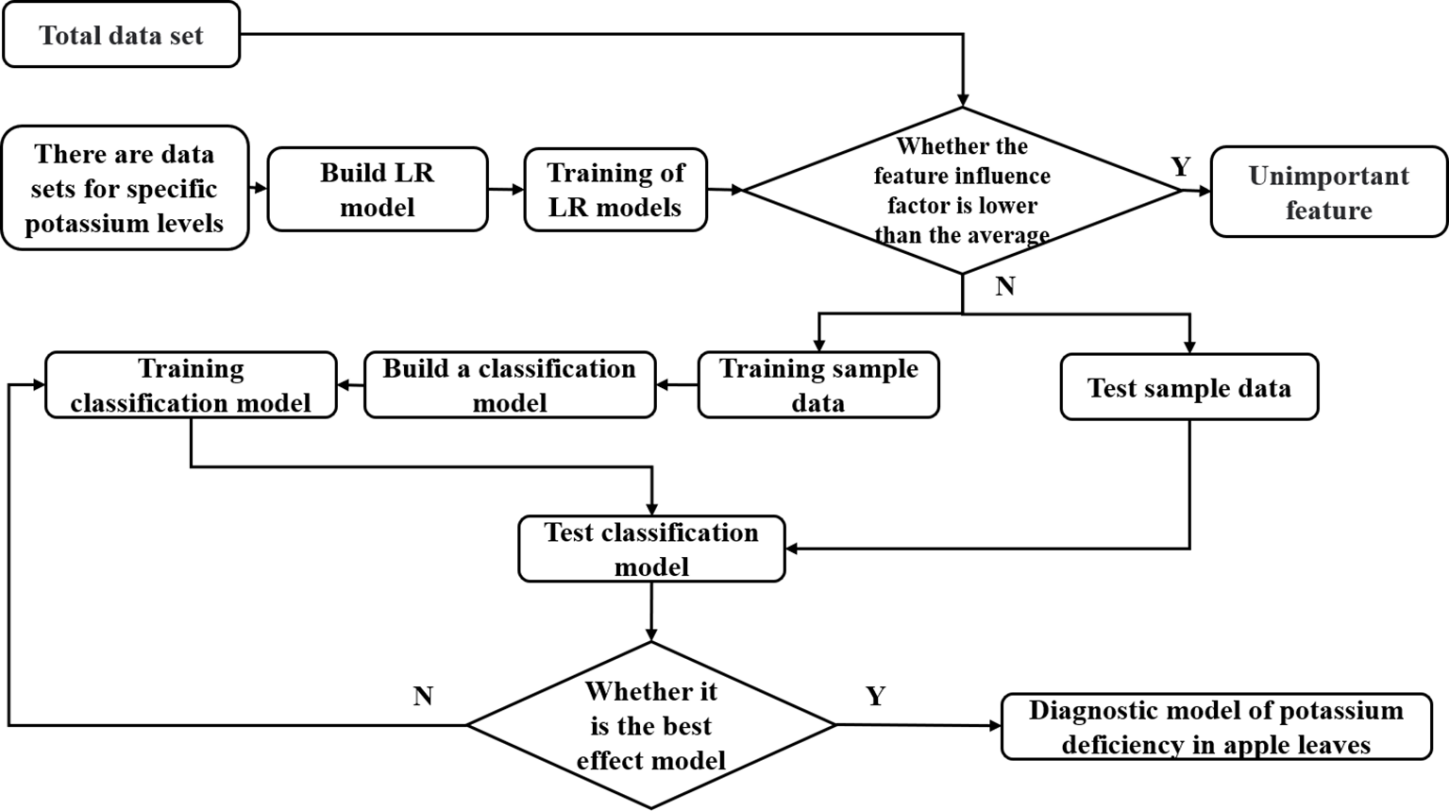
Method algorithm flow chart.

To reduce the complexity of the feature data and prevent issues such as overfitting, the features with an influence factor below 0.35 are deemed unimportant and removed from the dataset. Only the features G, B, L, A, S, V, and S_1_ are retained, and a new dataset is established. The constructed classification method algorithm is then trained using this data set, and the flowchart is designed (**Figure 9**).

**Figure 10:**
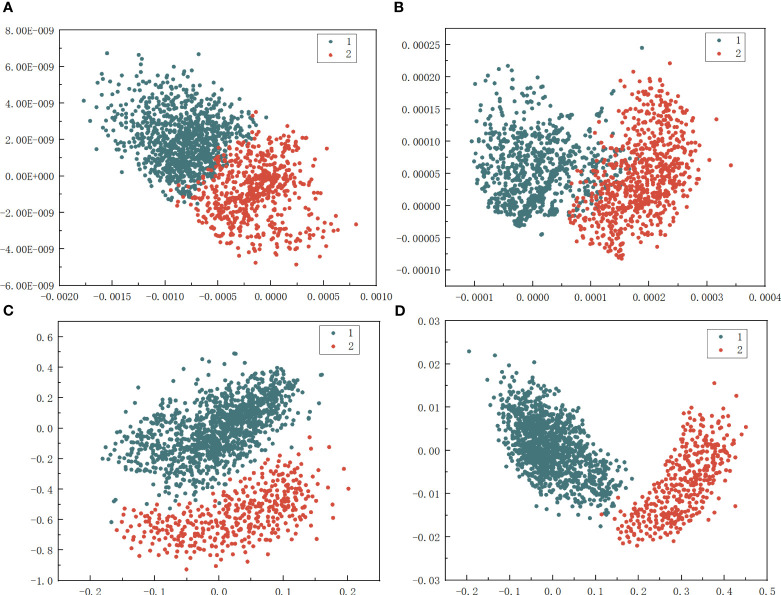
Fractal Visualization Graph of Features after LDA. **(A)** Flowering period. **(B)** Young fruit, period. **(C)** Fruit enlarging period. **(D)** Mature period.

In the implemented classification algorithm, the first step involved the application of Multiple Linear Regression (MLR) to screen the data, removing feature data with impact factors lower than 0.35. The remaining feature data was retained as samples for subsequent training. To further reduce the dimension of the data while retaining the category information of the feature data, the LDA method was employed to reduce the dimension of the sample data after removing the data. The optimal shape and color feature data of apple leaves in each growth period were obtained as the fundamental shape and color combination feature factor. Potassium-deficient leaves were labeled class 1, and regular leaves were labeled class 2. The characteristics of each growth period after LDA treatment were visualized (**Figure 10**).

Indeed, as depicted in **Figure 10**, it is evident that the data features processed by LDA exhibit high dispersion for different types of features, and the discrimination of leaf features is more apparent.

### 3.2 Determination of the optimal diagnostic method

The two-dimensional features with high dispersion after MLR-LDA dimensionality reduction were employed as input parameters to train and evaluate the potassium deficiency diagnosis method. The potassium-deficient leaves were labeled type 1, and the regular leaves were labeled type 2. 70% of the randomly selected data were utilized as the training set to train the potassium deficiency diagnosis method. In comparison, the remaining 30% was designated as the test set to evaluate the method’s effectiveness.

#### 3.2.1 The method of diagnosis based on MLR-LDA-SVM

**Figure 11:**
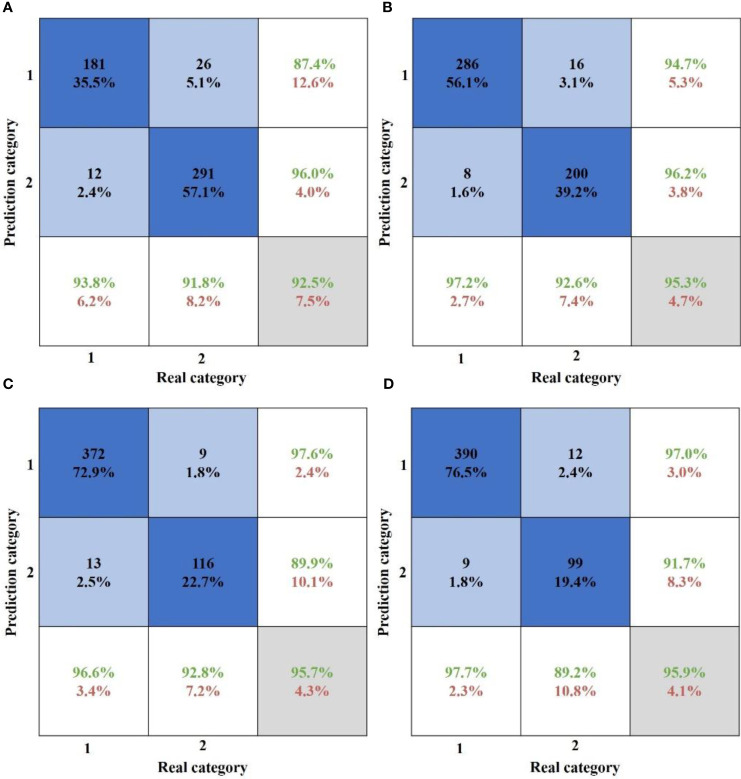
Confusion matrix of MLR-DA-SVM classification results at each growth period. **(A)** Flowering period. **(B)** Young fruit, period. **(C)** Fruit enlarging period. **(D)** Mature period.

This study employed 1190 samples processed by MLR-LDA as training samples to establish the SVM method. The penalty factor C of SVM is set to 10, and the RBF kernel width parameter is determined as 0.1 using the K-fold cross-validation method. The MLR-LDA-SVM diagnostic method was established for each growth period. A total of 510 test samples were utilized to evaluate the classification effectiveness of the MLR-LDA-SVM diagnostic method in each growth period. The confusion matrix displays the classification results (**Figure 11**).

#### 3.2.2 The method of diagnosis based on MLR-LDA-KNN

In this study, 1190 MLR-LDA samples were used as training samples to establish the KNN method. The parameter selection principle was based on the highest accuracy, and the number of nearest neighbor elements was set to 6, and the distance was calculated using the Manhattan Distance. Five hundred ten test samples were employed to examine the classification performance of the MLR-LDA-KNN diagnostic method in each growth period. The confusion matrix displays the classification results (**Supplementary Figure 3**).

#### 3.2.3 The method of diagnosis based on MLR-LDA-DT

In this study, 1190 MLR-LDA samples were used as training samples to establish the DT method. The parameter selection principle was based on the highest accuracy, and the size of the tree was limited by controlling the depth of the tree. The depth of the tree was set to 5, and the MLR-LDA-DT diagnostic method was established for each growth period. Five hundred ten test samples were employed to examine the classification effectiveness of the MLR-LDA-DT diagnostic method in each growth period. The confusion matrix displays the classification results (**Supplementary Figure 4**).

**Figure 12:**
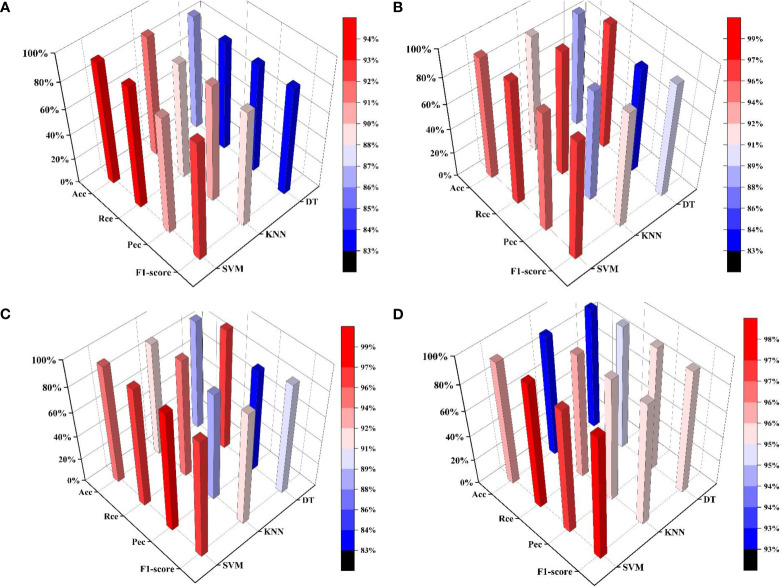
Comparison of classification results of different methods in different growth periods. **(A)** Flowering period. **(B)** Young fruit, period. **(C)** Fruit enlarging period. **(D)** Mature period.

After LDA processing, Acc, Rec, Pec, and f1 scores of SVM, KNN, and DT methods in different periods were compared (**Figure 12**).

Based on the comparison of the *Acc*, *Rec*, *Pec*, and *F1-score* results of different methods, the prediction accuracy of the MLR-LDA-SVM method was observed to be superior to that of the MLR-LDA-KNN and MLR-LDA-DT methods throughout the entire growth cycle of an apple, including the flowering period, young fruit period, fruit enlarging period, and mature period. Therefore, this study utilized the MLR-LDA-SVM-based diagnostic method for potassium deficiency in apple leaves to accurately diagnose whether apple leaves were potassium deficient at each growth period.

### 3.3 Field experiment and results

#### 3.3.1 Field experiment

##### 3.3.1.1 Test time and place

Leaf collection and image acquisition were conducted on April 26 (flowering period), June 16 (young fruit period), August 11 (Fruit enlarging period), and September 30 (mature period) in an unfertilized area of the third section of the Wanlin Technology Apple Base. The collection was conducted simultaneously with the collection of leaves in the training and test sets. Measurements of leaf potassium content in the third patch were made using flame photometry, and the data were saved for validation work after modeling.

##### 3.3.1.2 Experimental protocol

At each growth period, 20 representative red Fuji apple trees in the third plot were randomly selected, and leaf images were collected for the field experiment of apple trees’ potassium deficiency diagnosis method. After collecting leaf images, the flame photometry technique was employed to determine the potassium content in the leaves. This valuable information was utilized to improve the classification results of the experimental method. The preprocessing of leaf images, feature extraction, and other essential tasks for the validation set were carried out in parallel with those for the test and validation sets.

##### 3.3.1.3 Test index

The generalization ability and robustness of the method were evaluated by Acc.

#### 3.3.2 Field experiment results of different growth periods

The MLR-LDA-SVM method had 17 errors in diagnosis results for 200 verifier blade images during the flowering period(**Supplementary Figure 5**), with an accuracy rate of 91.5%.

The MLR-LDA-SVM method had 12 errors in diagnosis results for 200 validation set leaf images at the young fruit period (**Supplementary Figure 6**), with an accuracy rate of 94%.

The MLR-LDA-SVM method had 13 errors in the diagnosis results of 200 validation set blade images in the fruit enlarging period (**Supplementary Figure 7**), and the accuracy rate was 93.5%.

The MLR-LDA-SVM method has ten errors in diagnosis results for 200 validation set blade images at the mature period (**Supplementary Figure 8**), and the accuracy rate is 95%.

## 4 Conclusions

This study collected potassium-deficient and regular apple leaves samples at four distinct growth stages through targeted fertilizer interventions on selected apple trees. Digital image processing techniques were used to extract a comprehensive set of 12 color features and 10 shape features from individual leaf samples to capture the color and other specific symptom manifestations associated with potassium deficiency. A rigorous screening process was implemented using linear regression to ensure data integrity and reduce dimensionality. Features with an impact factor below 0.35 were considered non-essential and eliminated. The remaining features underwent linear discriminant analysis to reduce dimensionality and optimize the most influential characteristics. This meticulous approach aimed to improve classification accuracy, minimize variables, and compress the feature space, ultimately extracting the key factors crucial for diagnosing potassium deficiency at each growth stage. After comparative method analysis, the MLR-LDA-SVM method was identified as the most suitable diagnostic framework for detecting potassium deficiency in apple trees. Field experiments were conducted to validate the method’s performance, revealing an impressive average accuracy of 93.5% throughout the growth cycle. The study showcases how the method’s strength and exceptional generalization abilities enable the precise identification of potassium deficiency in apple leaves, regardless of the growth stages. The goal is to enhance the accuracy of leaf potassium deficiency diagnosis while simultaneously lowering the diagnostic cost. Furthermore, expanding the database in the subsequent use process, continuously strengthening the diagnostic model, and providing theoretical guidance for intelligent and precise water and fertilizer management in orchards are critical for achieving the desired outcomes.

## Data availability statement

The raw data supporting the conclusions of this article will be made available by the authors, without undue reservation.

## Author contributions

KX: Conceptualization, Data curation, Writing – original draft. L-LS: Conceptualization, Supervision, Writing – original draft, Writing – review & editing. JW: Data curation, Investigation, Methodology, Writing – review & editing. S-XL: Funding acquisition, Investigation, Methodology, Writing – review & editing. H-WY: Conceptualization, Formal Analysis, Investigation, Methodology, Software, Writing – review & editing. NX: Conceptualization, Investigation, Methodology, Software, Writing – review & editing. J-XW: Formal Analysis, Funding acquisition, Methodology, Supervision, Validation, Writing – review & editing. H-JZ: Funding acquisition, Resources, Software, Supervision, Validation, Visualization, Writing – review & editing.

## References

[B1] Chen H. B. Li T. (2018). Common deficiency disease and its control in apple trees. Chin. Fruit Vegetable 38 (06), 57–59. doi: 10.19590/j.cnki.1008-1038.2018.06.018

[B2] Etemadi S. Khashei M. (2021). Etemadi multiple linear regression. Measurement 186, 12. doi: 10.1016/j.measurement.2021.110080

[B3] Fuentes-Alventosa A. Gomez-Luna J. Medina-Carnicer R. (2022). GUD-Canny: a real-time GPU-based unsupervised and distributed Canny edge detector. J. Real-Time Image Process 19 (3), 591–605. doi: 10.1007/s11554-022-01208-0

[B4] Furlanetto R. H. Rafael M. N. Guilherme T. C. L. Silva G. F. C. Junior A. d. O.. (2021). Identification and quantification of potassium (K+) deficiency in maize plants using an uncrewed aerial vehicle and visible / near-infrared semi-professional digital camera. Int. J. Remote Sens. 42 (23), 8783–8804. doi: 10.1080/01431161.2020.1871091

[B5] Gao Z. H. Gu C. F. Yang J. H. Gao S. S. Zhong Y. M. (2020). Random weighting-based nonlinear gaussian filtering. IEEE Access 8, 19590–19605. doi: 10.1109/access.2020.2968363

[B6] Gardner-Lubbe S. (2021). Linear discriminant analysis for multiple functional data analysis. J. Appl. Stat. 48 (11), 1917–1933. doi: 10.1080/02664763.2020.1780569 35706433 PMC9042036

[B7] Guenther N. Schonlau M. (2016). Support vector machines. Stata J. 16 (4), 917–937. doi: 10.1177/1536867x1601600407

[B8] Jae-Won C. Trung T. T. Huynh Thien T. L. Geon-Soo P. Van Dang C. Jong-Wook K. (2018). “A nutrient deficiency prediction method using deep learning on development of tomato fruits,” in International Conference on Fuzzy Theory and Its Applications (iFUZZY), Daegu, Korea (South), 338–341. doi: 10.1109/iFUZZY.2018.8751688

[B9] Jiang J. Y. Tsai S. C. Lee S. J. (2012). FSKNN: Multi-label text categorization based on fuzzy similarity and k nearest neighbors. Expert Syst. Appl. 39 (3), 2813–2821. doi: 10.1016/j.eswa.2011.08.141

[B10] Korkmaz M. (2021). A study over the general formula of regression sum of squares in multiple linear regression. Numer. Meth. Part Differ. Equ. 37 (1), 406–421. doi: 10.1002/num.22533

[B11] Li J. H. Zhu L. L. Song S. Y. (2022). Application of digital image technology in nitrogen nutrition diagnosis of Chinese cabbage. Northeast Agric. Sci. 47 (02), 129–133. doi: 10.16423/j.CNCHI.1003-8701.2022.02.028

[B12] Luo J. J. Yang H. Y. Lu Y. Yi L. Sun A. Z. (2020). It was based on a genetic algorithm to optimize the BP neural network to diagnose rice nitrogen nutrition. China's Agric. Sci. Technol. leader 22 (8), 83–92. doi: 10.13304/j.ykjdb.2019.1058

[B13] Merchant M. Paradkar V. Khanna M. Gokhale S. (2018). “Mango leaf deficiency detection using digital image processing and machine learning,” in 2018 3rd International Conference for Convergence in Technology (I2CT), Pune, India, 1–3. doi: 10.1109/I2CT.2018.8529755

[B14] Peng H. X. Wei X. Y. Xiao Y. X. Sun Y. Shang S. P. Biggs A. R.. (2015). Management of Valsa canker on apple with adjustments to potassium nutrition. Plant Dis. 100, 884–889. 10.1094/PDIS-09-15-0970-RE 30686143

[B15] Qi H. (2017). Hyperspectral Based Potassium Nutrition Monitoring of Wheat (Nanjing Agricultural University).

[B16] Rangel B. M. S. Fernández M. A. A. Murillo J. C. Ortega J. C. P. Arreguín J. M. R. (2016). KNN-based image segmentation for grapevine potassium deficiency diagnosis. Communications Comput. Cholula, Mexico 2016, 48–53. doi: 10.1109/CONIELECOMP.2016.7438551

[B17] Shi Y. Y. (2011). Diagnosis and modeling of nitrogen, phosphorus and potassium nutrition in rice based on digital images. Learned scholar, Zhejiang University.

[B18] Shi J. Li Y. Zhai X. D. Guo Z. M. Zou X. B. (2019). Nondestructive diagnostics of magnesium deficiency based on distribution features of chlorophyll concentrations map on cucumber leaf. J. Plant Nutr. 20, 2773–2783. doi: 10.1016/j.biosystemseng.2021.11.001

[B19] Shi J. Y. Wang Y. Y. Li Z. H. Huang X. W. Shen T. T. Zou X. B. (2021). Simultaneous and nondestructive diagnostics of nitrogen/magnesium/potassium-deficient cucumber leaf based on chlorophyll density distribution features. Biosyst. Eng. 212, 458–67. doi: 10.1080/01904167.2019.1659332

[B20] Shu H. R. Zhang S. Z. (2021). The 70 years’ development and the prospect of the apple industry in China. Deciduous Fruits. 1 (53), 01–03. doi: 10.13855/j.cnki.legs.2021.01.001

[B21] Takehisa H. Ando F. Takara Y. Ikehata A. Sato Y. (2022). Transcriptome and hyperspectral profiling allow assessing phosphorus nutrient status in rice under field conditions. Plant Cell Environ. 45 (5), 1507–1519. doi: 10.1111/pce.14280 35128701

[B22] Tian Y. F. (2022). Apple cultivation techniques and nutritional diagnostic methods for apple. Agric. Dev. Equip. 03), 202–204.

[B23] Ullah R. Abbas Z. Bilal M. Habib F. Iqbal J. Bashir F.. (2022). Method development and validation for the determination of potassium (K2O) in fertilizer samples by flame photometry technique. J. King Saud Univ. - Sci. 34 (5). doi: 10.1016/j.jksus.2022.102070

[B24] Wang C. (2023). Analysis of rural land pollution and soil environmental protection methods. Agric. Technol. Equip. 01), 75–76.

[B25] Wang J. B. Lu K. Xue J. He N. Shao L. (2018). Single image dehazing based on the physical method and MSRCR algorithm. IEEE Trans. Circuits Syst. Video Technol. 28 (9), 2190–2199. doi: 10.1109/tcsvt.2017.2728822

[B26] Wang X. D. Shi M. Y. Zhang Y. Chen S. J. (2019). Potassium content and control of apple rot in Tacheng area. Agric. Jilin 13, 73. doi: 10.14025/j.cnki.jlny.2019.13.034

[B27] Wang F. Wang Q. Nie F. P. Li Z. H. Yu W. Z. Ren F. J. (2020). A linear multivariate binary decision tree classifier based on K-means splitting. Pattern Recognit 107, 13. doi: 10.1016/j.patcog.2020.107521

[B28] Wei T. L. Yang S. D. Cheng S. P. Pei M. S. Liu H. N. Yu Y. H.. (2022). Transcriptome analysis reveals the responsive pathways to potassium (K+) deficiency in the roots and shoots of grapevines. Scientia Hortic., 293. doi: 10.1016/j.scienta.2021.110742

[B29] Xu T. Y. Bai J. C. Guo Z. H. Jin Z. Y. Yu F. H. (2023). Uncrewed aerial vehicle hyperspectral remote sensing method for nitrogen nutrition diagnosis in rice. Trans. Chin. Soc. Agric. Machinery 54 (02), 189–197+222. https://kns.cnki.net/kcms/detail/11.1964.s.20230105.1638.006.html.

[B30] Yang F. Gao X. Y. Li H. L. Y M. Shao S. L. (2021). Detection of potassium content in grape leaves based on image processing and deep learning. Forestry Machinery Woodworking Equip. 49 (02), 9–15+21. doi: 10.13279/j.carolcarrollski

[B31] Zhang K. Zhang A. Li C. (2016). Nutrient deficiency diagnosis method for rape leaves using color histogram on HSV space. Transactions of the Chinese Society of Agricultural Engineering 32, 179–187

[B32] Zhang W. D. Dong L. L. Pan X. P. Zhou J. C. Qin L. Xu W. H. (2019). Single image defogging based on multi-channel convolution MSRCR. IEEE Access 7, 72492–72504. doi: 10.1109/access.2019.2920403

[B33] Zheng X. Q. (2022). Application of image processing in agriculture: A case study of apple leaf diseases based on LeNet-5 method. Nanfang Agric. Machinery. 53 (15), 36–39.

[B34] Zheng S. Ding C. Nie F. P. Huang H. (2019). Harmonic mean linear discriminant analysis. IEEE Trans. Knowl. Data Eng. 31 (8), 1520–1531. doi: 10.1109/tkde.2018.2861858

[B35] Zhou X. Yang M. Chen X. Ma L. Yin C. Qin S. (2023). Estimation of cotton nitrogen content based on multi-angle hyperspectral data and machine learning models. Remote Sens. 15, 955. doi: 10.3390/rs15040955

