# Supplementary figures and images for: Potassium deficiency diagnosis method of apple leaves based on MLR-LDA-SVM
Source: Front Plant Sci. 2023 Nov 29;14:1271933. doi: 10.3389/fpls.2023.1271933 (PMC10716244; doi:10.3389/fpls.2023.1271933)

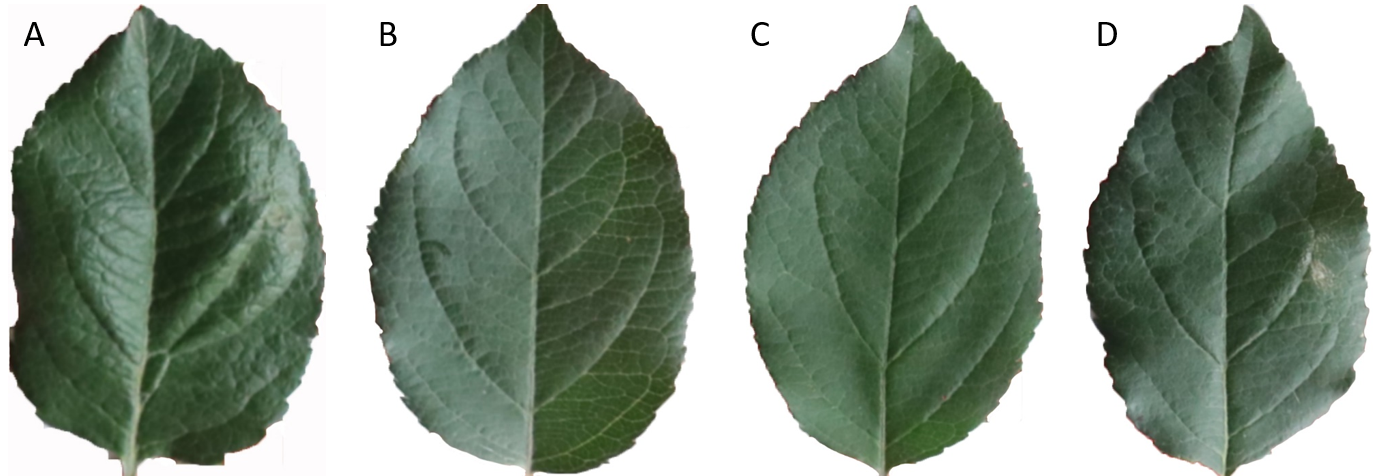

Supplement: Supplementary file 3 [file Image_1.tif]

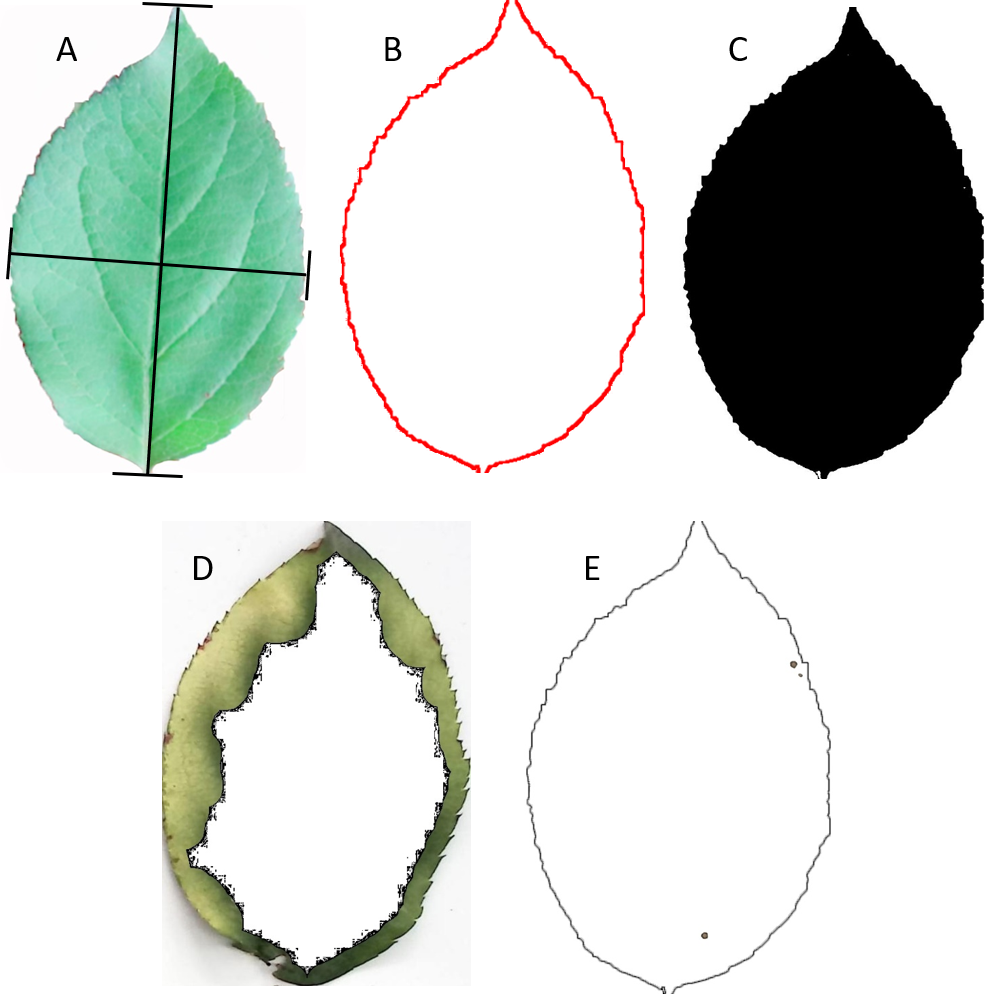

Supplement: Supplementary file 4 [file Image_2.tif]

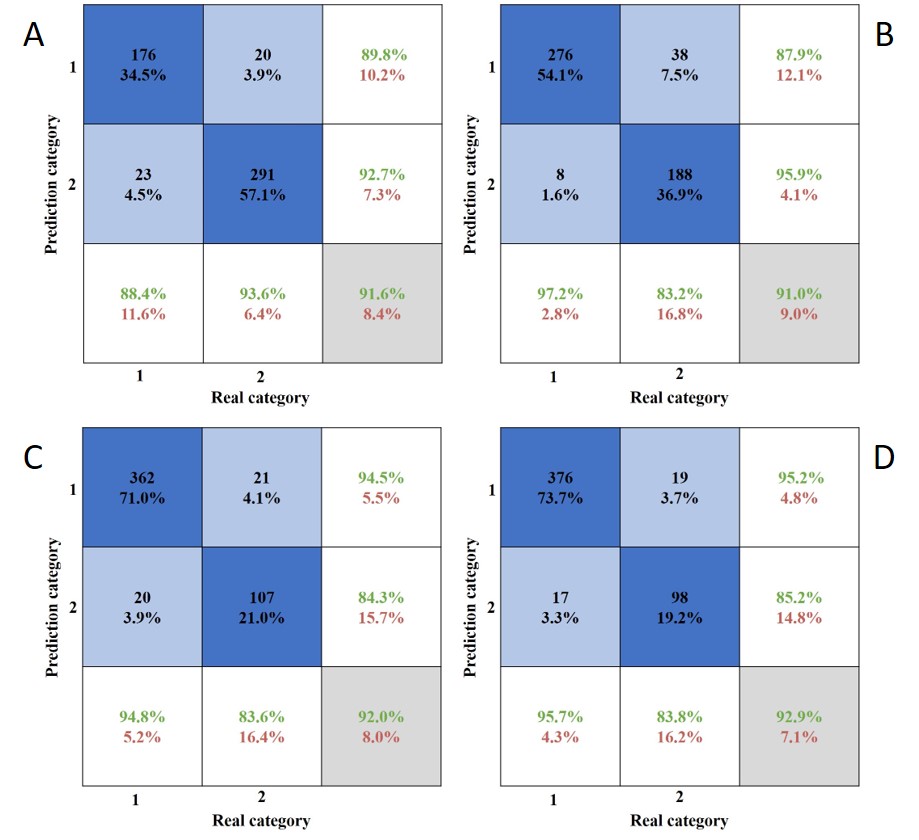

Supplement: Supplementary file 5 [file Image_3.jpeg]

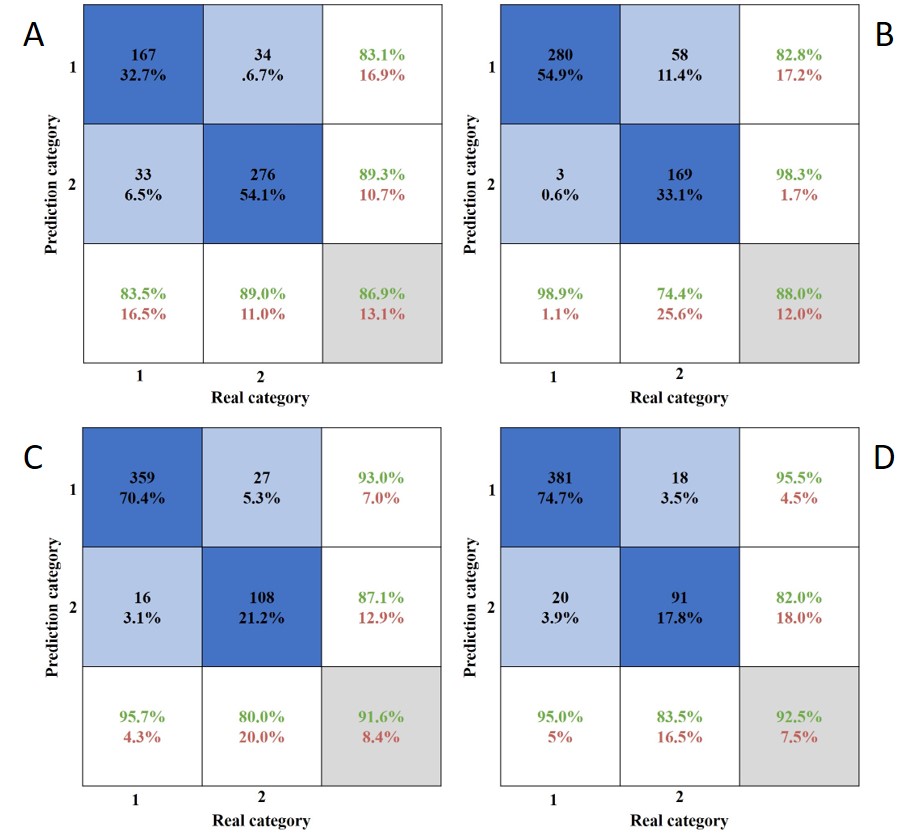

Supplement: Supplementary file 6 [file Image_4.jpeg]

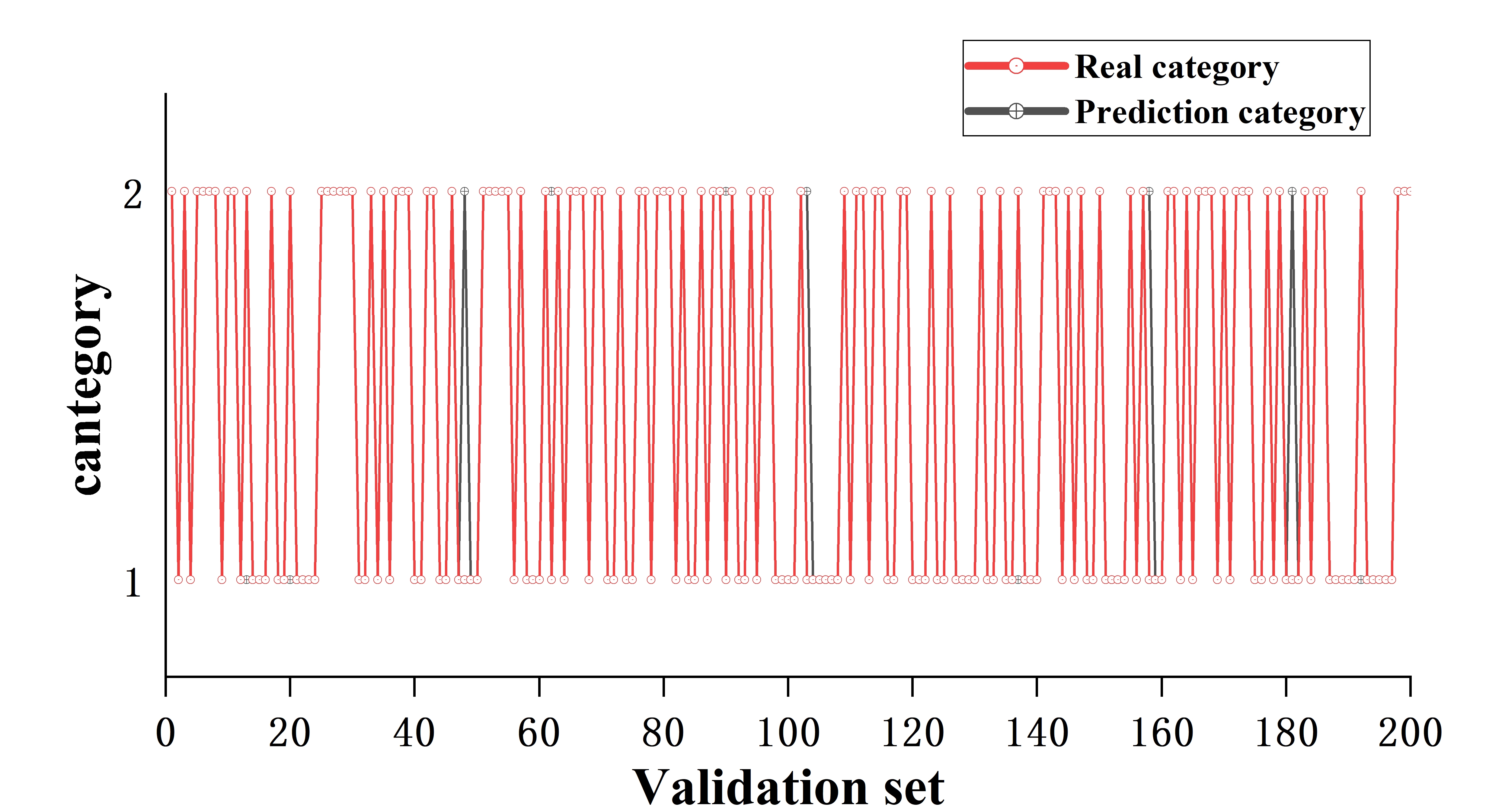

Supplement: Supplementary file 7 [file Image_5.jpeg]

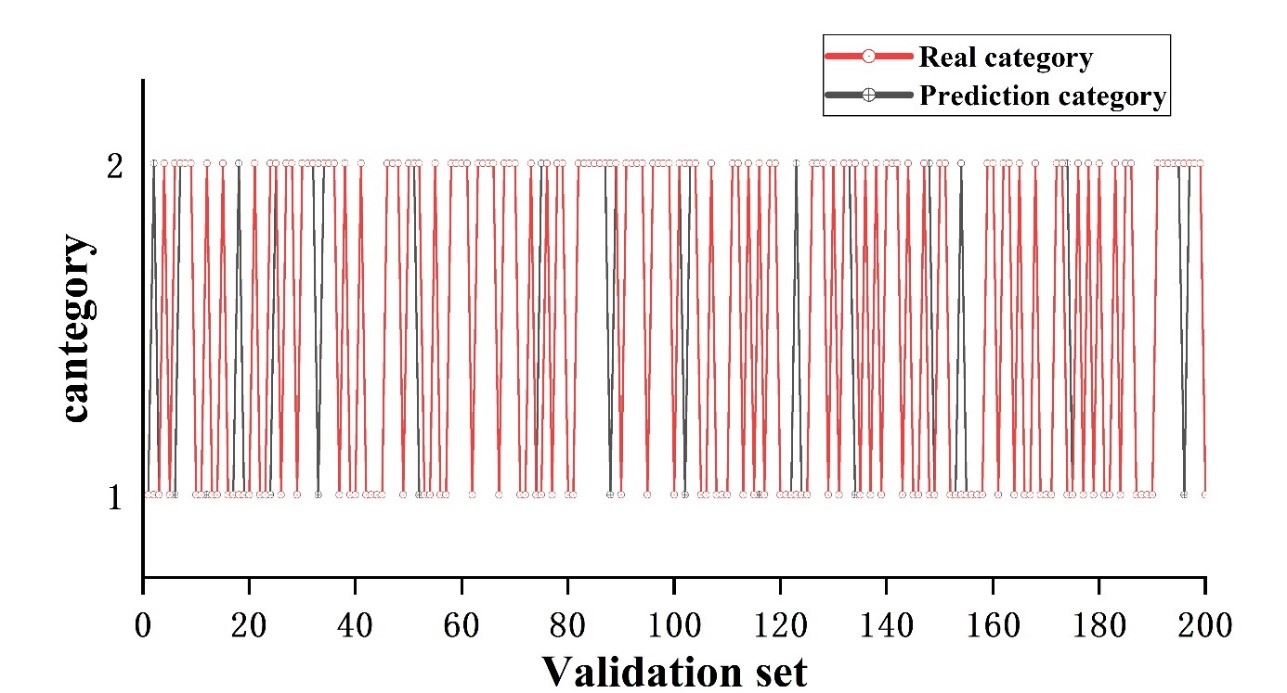

Supplement: Supplementary file 8 [file Image_6.jpeg]

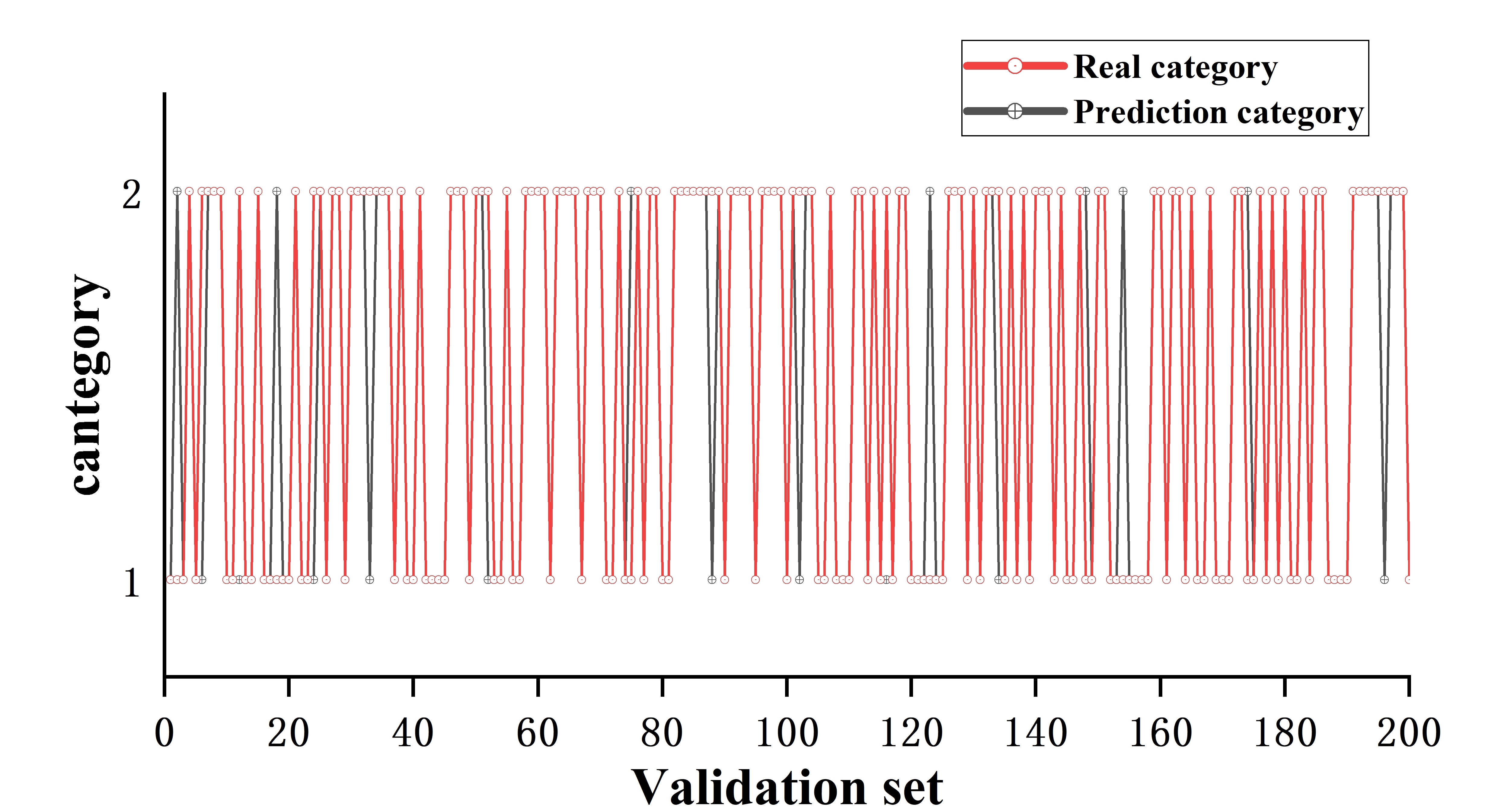

Supplement: Supplementary file 9 [file Image_7.jpeg]

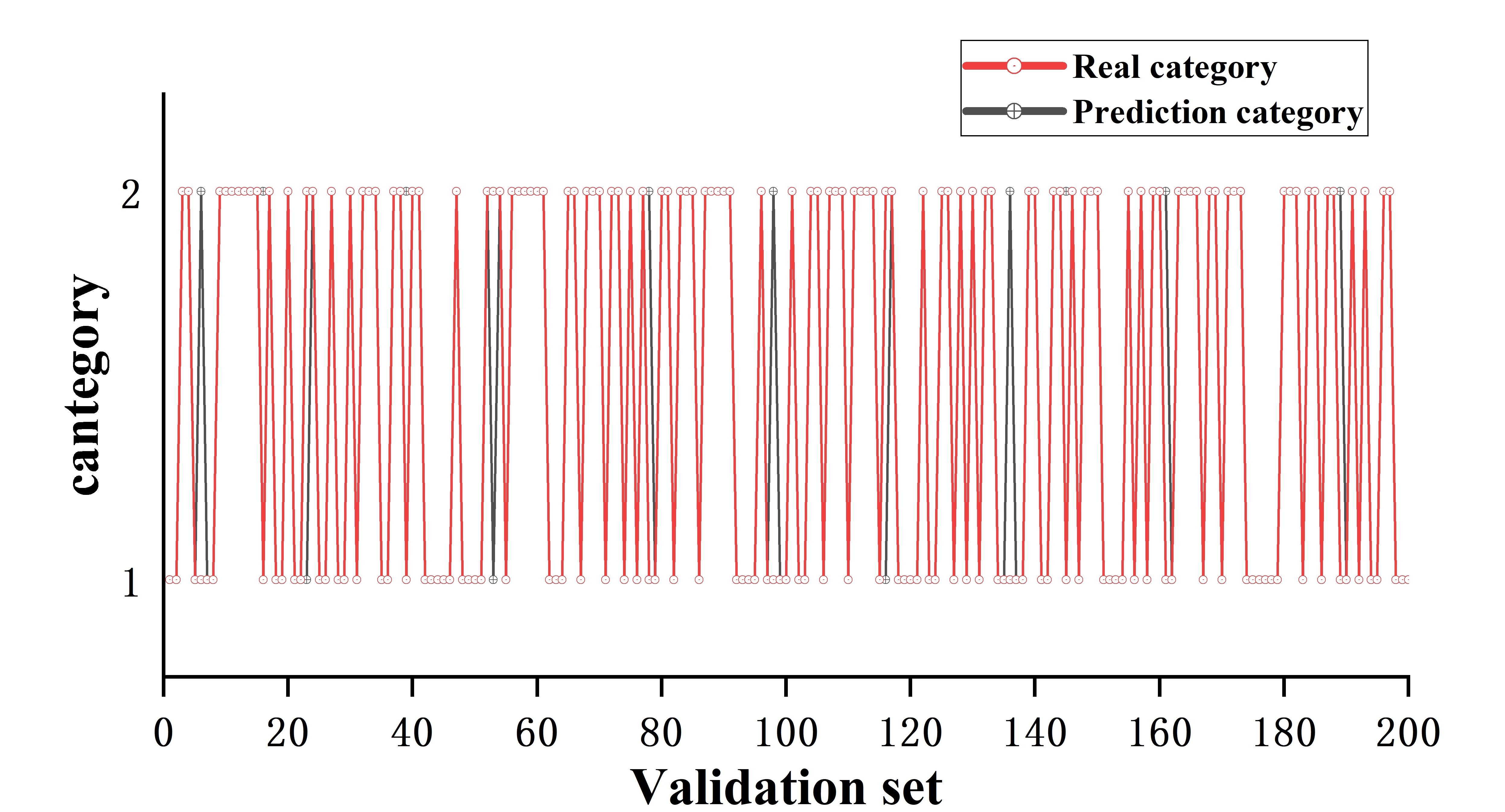

Supplement: Supplementary file 10 [file Image_8.jpeg]
